# Factors associated with the lethality of human leptospirosis in Brazil

**DOI:** 10.1590/0102-311XEN144324

**Published:** 2025-07-18

**Authors:** Alice Nardoni Marteli, Laurindo Antonio Guasselli, Décio Diament, Daniel Savignon Marinho, Geraldo Marcelo da Cunha, Federico Costa

**Affiliations:** 1 Programa de Pós-graduação em Sensoriamento Remoto, Universidade Federal do Rio Grande do Sul, Porto Alegre, Brasil.; 2 Instituto de Infectologia Emílio Ribas, São Paulo, Brasil.; 3 Escola Nacional de Saúde Pública Sergio Arouca, Fundação Oswaldo Cruz, Rio de Janeiro, Brasil.; 4 Centro de Pesquisas Gonçalo Moniz, Fundação Oswaldo Cruz, Salvador, Brasil.

**Keywords:** Leptospirosis, Mortality, Sociodemographic Factors, Leptospirose, Mortalidade, Fatores Sociodemográficos, Leptospirosis, Mortalidad, Factores Sociodemográficos

## Abstract

Human infection by *Leptospira* results from direct exposure or indirect contact with soil or water contaminated by the urine of carrier mammals. Despite the current knowledge about the modes of infection, there are still gaps in understanding the factors that contribute to the disease lethality. Aiming to identify factors associated with death from leptospirosis in Brazil, a retrospective exploratory cross-sectional study was conducted using data from the Brazilian Information System for Notificable Diseases of the Brazilian Ministry of Health, from 2007 to 2019. From an initial total of 50,640 confirmed cases, 38,206 (75.45%) cases were selected for analysis, of which 10.39% (3,968) represented deaths. Among the sociodemographic factors associated with death from leptospirosis, notable ones included contact with trash/rubble, work-related infection, male gender, non-white skin color, and increased risk with age. Among the clinical factors, notable ones included the presence of respiratory alterations, clinical-epidemiological confirmation criteria, pulmonary hemorrhage, kidney failure, jaundice, cardiac alterations, and vomiting. Regarding geographical distribution, residing in the Southeast increased the chance of death by 83% compared to individuals residing in the North, which had the lowest proportion of deaths (5.68%). The results found in this study provide an overview of the lethality of leptospirosis in humans in Brazil. Regional differences should be better investigated to guide health policies.

## Abstract (pt)

A infecção humana por *Leptospira* resulta da exposição direta ou contato indireto com solo ou água contaminada pela urina de mamíferos portadores. Apesar do conhecimento atual sobre os modos de infecção, ainda existem lacunas na compreensão dos fatores que contribuem para a letalidade da doença. Para identificar os fatores associados ao óbito por leptospirose no Brasil, foi realizado um estudo transversal exploratório retrospectivo utilizando dados do Sistema de Informação de Agravos de Notificação do Ministério da Saúde, no período de 2007 a 2019. De um total inicial de 50.640 casos confirmados, foram selecionados para análise 38.206 (75,45%) casos, dos quais 10,39% (3.968) representaram óbitos. Dentre os fatores sociodemográficos associados ao óbito por leptospirose, destacam-se o contato com lixo/entulho, infecção relacionada ao trabalho, sexo masculino, raça/cor não branca e aumento do risco com a idade. Dentre os fatores clínicos, destacaram-se a presença de alterações respiratórias, critérios de confirmação clínico-epidemiológica, hemorragia pulmonar, insuficiência renal, icterícia, alterações cardíacas e vômitos. Em relação à distribuição geográfica, residir na Região Sudeste aumentou a chance de óbito em 83% em relação aos indivíduos residentes na Região Norte, que apresentaram a menor proporção de óbitos (5,68%). Os resultados encontrados neste estudo fornecem uma visão geral da letalidade da leptospirose em humanos no Brasil. As diferenças regionais devem ser melhor investigadas para orientar as políticas de saúde.

## Abstract (es)

La infección humana por *Leptospira* se contrae por la exposición directa o el contacto indirecto con el suelo o el agua contaminados por la orina de mamíferos portadores. A pesar del conocimiento actual sobre los modos de transmisión, todavía existen lagunas en la comprensión de los factores que contribuyen a la letalidad por esta enfermedad. Para identificar los factores asociados con la muerte por leptospirosis en Brasil, se realizó un estudio transversal exploratorio retrospectivo a partir de los datos del Sistema de Información de Agravios de Notificación del Ministerio de Salud de Brasil, en el período de 2007 a 2019. De un total inicial de 50.640 casos confirmados, se seleccionaron 38.206 (75,45%) casos para el análisis, de los cuales el 10,39% (3.968) progresaron a muertes. Entre los factores sociodemográficos asociados a la muerte por leptospirosis, destaca el contacto con basura/residuos, infección laboral, sexo masculino, raza/color no blanco y mayor riesgo con la edad. Entre los factores clínicos se destacó la presencia de alteraciones respiratorias, criterios de confirmación clínica y epidemiológica, hemorragia pulmonar, insuficiencia renal, ictericia, alteraciones cardíacas y vómitos. En cuanto a la distribución geográfica, vivir en la Región Sureste aumentó la probabilidad de muerte en un 83% en comparación con las personas que viven en la Región Norte y que tuvieron la menor proporción de muertes (5,68%). Los resultados encontrados en este estudio proporcionan una visión general de la letalidad de la leptospirosis en humanos en Brasil. Las diferencias regionales necesitan investigarse más a fondo para guiar las políticas de salud.

## Introduction

Leptospirosis is a neglected waterborne zoonosis, primarily transmitted via exposure to environments contaminated with rat urine, as these animals serve as reservoirs for the bacteria *Leptospira* spp. ^1^. This disease incidence is higher in tropical countries in which contact with rodents in urban and rural areas significantly increases the risk of infection. The risk of infection also increases with proximity to residential environments near vacant lots, trash accumulation, septic tanks, grease traps, and sewage systems ^2^. Additionally, there is an increased risk for various occupations, such as veterinarians, slaughterhouse workers, urban cleaners, sewage treatment plant operators, and those involved in agricultural activities ^2^.

After initial exposure to the bacteria, individuals may have a range of symptoms, influenced by the specific serovar of the infecting *Leptospira*, the individual’s immune status, the bacterial load, and access to reference health facilities. Symptoms can range from mild - with characteristics similar to the flu - to severe conditions including pulmonary, kidney and liver failure, culminating in the classic Weil’s disease, which can be fatal ^3^. Factors such as age, patient’s immune health, and the intensity and frequency of exposure determine risk severity ^3^ ^,^ ^4^. Additionally, symptoms such as fever, myalgia, headache, calf pain, vomiting, diarrhea, jaundice, and respiratory and cardiac alterations have been associated with increased disease lethality in various geographic contexts, including India ^5^, Australia ^6^, Malaysia ^1^, and Brazil ^7^.

The similarity of these symptoms with other tropical diseases, such as dengue and chikungunya fever, often hinders accurate diagnosis, contributing to the underreporting of cases and inadequate treatment ^2^ ^,^ ^8^. Symptoms like calf pain, conjunctival congestion, and pulmonary hemorrhage may assist health professional discriminate leptospirosis from other febrile tropical diseases ^1^ ^,^ ^9^. Despite the World Health Organization (WHO) not including it on its list, the Public Library of Science (PLOS) considers leptospirosis a neglected tropical disease ^10^. 

A systematic review ^8^ analyzing 80 studies conducted in 34 countries estimated that there are 1.03 million cases of leptospirosis and 58,900 associated deaths annually. In Brazil, leptospirosis was reported in nearly half of the municipalities, with 2,600 of the 5,570 municipalities reporting confirmed cases between 2007 and 2017, covering all regions of Brazil. This resulted in an average of 3,846 cases per year and an incidence of approximately 1.9 per 100,000 inhabitants ^11^. The global burden of leptospirosis, measured in disability-adjusted life years (DALYs), is estimated at 2.90 million DALYs lost annually. These figures highlight the high lethality and significant impact of the disease regarding morbidity and mortality ^8^ ^,^ ^12^.

The current landscape of studies on leptospirosis reveals a significant gap in knowledge regarding access to healthcare services, early diagnosis, and the consequent prognosis of the disease. Notably, few studies focused on the factors associated with prolonged hospitalization and lethality due to leptospirosis. In a study conducted in the French West Indies, the mean interval between hospitalization and death of patients with leptospirosis was estimated to be 8 days ± 7 days ^5^, potentially extending up to 50 days, while the period before hospital admission can last up to 30 days ^1^.

This study aims to identify significant factors associated with death in patients diagnosed with leptospirosis using the Brazilian Information System for Notificable Diseases (SINAN, acronym in Portuguese) in Brazil from 2007 to 2019. By elucidating these determinants, it is hoped that this study can contribute to the optimization of strategies that may reduce the lethality associated with leptospirosis in Brazil.

## Material and methods

### Data source and study design

This is a cross-sectional study analysis based on secondary data, targeting confirmed cases of leptospirosis in Brazil. The data were sourced from the SINAN of the Brazilian Health Informatics Department (DATASUS, acronym in Portuguese) ^13^ for the period between 2007 and 2019. The variable of interest was the evolution of cases, specifically whether the patient survived or died within 90 days of the diagnosis. By considering this time frame, 98.96% of all deaths were accounted for. It was suggested that deaths occurring beyond this period may be related to other causes. 

**Figure 1:**
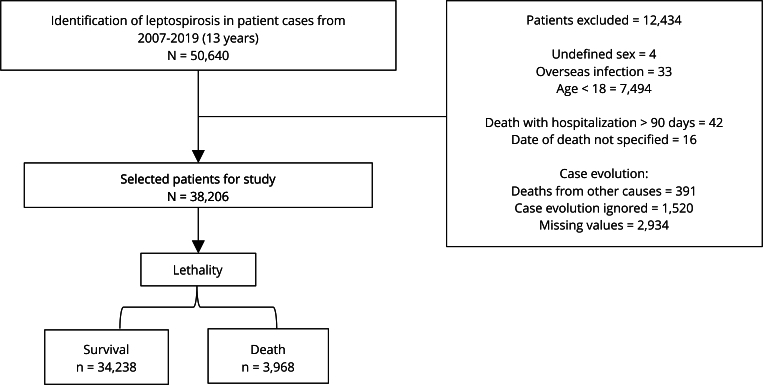
Study flowchart.

Of the total 50,640 notified cases, exclusions were made based on the following criteria: patients with undefined sex (n = 4), infection abroad (n = 33), under 18 years old (n = 7,494), deaths with hospitalization exceeding 90 days (n = 42), and unspecified date of death (n = 16). Cases with different disease outcomes (n = 391), those ignored (n = 1,520), and records with missing values (n = 2,934) were also excluded. After applying the exclusion criteria, the final study population comprised 38,206 patients. From this population, the lethality of the disease was evaluated, identifying 34,238 survival cases and 3,968 deaths (Figure 1).

### Study variables

**Figure 2:**
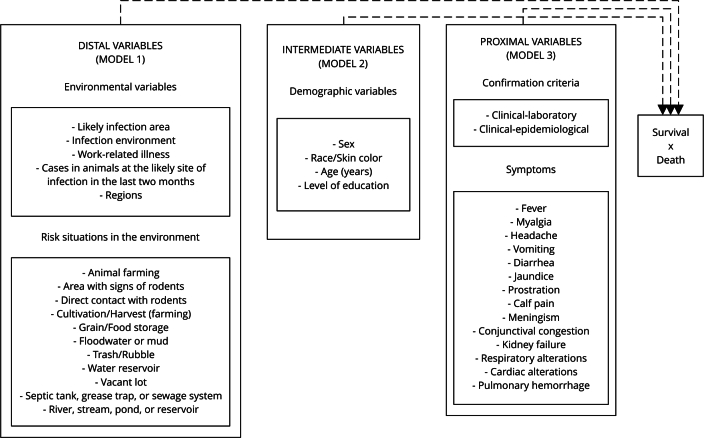
Description of the independent variables according to the hierarchical model.

Initially, 35 variables from the notification form ^13^ were selected and classified into three hierarchical levels as shown in Figure 2. Hierarchical analysis has been used in epidemiological studies to elucidate risk factors associated with health-related conditions ^14^ ^,^ ^15^. This type of analysis incorporates different hierarchical levels of determination concerning an outcome ^15^. The advantage of this approach lies in its ability to control for confounding effects among variables, enabling a clearer interpretation of the relationships at each level. It starts with distal variables, typically related to socioeconomic and environmental contexts, and progresses to proximal variables, such as behavioral and biological factors, reflecting a more precise understanding of the mechanisms linking social determinants to health outcomes ^16^.

In this study, the distal variables were related to the environment and included the probable area of infection, differentiating urban and rural settings, the context of the infection (home, work, leisure), the association with occupational activities, the presence of animal cases at the probable infection site in the past two months, and risk exposure in the 30 days preceding the onset of symptoms (variables in the notification form that consider the incubation period of the disease). These risk situations include activities such as animal husbandry, contact with areas infested with rodents, direct contact with rodents, staying in vacant lots, crops/harvesting areas, interaction with grain/food storage, exposure to floodwater or mud, handling of trash/rubble, access to water tanks, septic tanks, grease traps, or sewage systems, and contact with bodies of water such as rivers, streams, ponds, or reservoirs. The state to which the patients belonged were categorized and grouped according to the macroregions of Brazil. The intermediate variables were demographic and included patients’ sex, skin color, age, and education level. The proximal variables pertained to clinical aspects and included the criteria for disease confirmation, whether clinical-laboratory or clinical-epidemiological, as well as the signs and symptoms patients presented, namely: fever, myalgia, meningism, headaches, conjunctival congestion, calf pain, vomiting, diarrhea, jaundice, kidney failure, respiratory alterations, prostration, cardiac alterations, and pulmonary hemorrhage.

### Statistical analysis

Incidence and lethality rates, categorized according to the distribution of their respective quartiles, were calculated by states and presented in thematic maps. Incidence was estimated per 100,000 inhabitants, using the annual population estimates for each state from 2007 to 2019 ^13^ as the denominator. Lethality was estimated by multiplying the ratio of the number of deaths to the total number of confirmed cases by 100 ^17^.

Due to the presence of significant missing values in the database, data imputation was performed. This process aims to replace missing values with plausible estimates, enabling a complete analysis of the dataset. Data imputation is crucial for maintaining the integrity and utility of the dataset, preventing bias that may arise from excluding cases with missing data ^18^ ^,^ ^19^. Among the existing methods, multivariate imputation by chained equations (MICE) stand out for its flexibility and ability to handle the complexity of the data. This method performs multiple imputations, considering the joint distribution of the variables ^20^ ^,^ ^21^.

The proportions of deaths associated with each variable were calculated, and the association with death was compared using the chi-square test to determine the statistical significance of the observed differences. Logistic regression models were used to estimate the odds ratio (OR) for death resulting from the disease.

For variable selection within each level, we adopted the stepwise backward selection criterion, in which all potentially important variables are initially included in the model, and those less significant are sequentially removed based on a p-value < 0.15 for retention in the model. Therefore, in the final variable selection process within each level, a stepwise selection approach was used, considering the imputation of variables with the *miceafter* package (https://cran.r-project.org/web/packages/miceafter/index.html). All statistical analyses were performed using the R programming language (http://www.r-project.org), while map generation was carried out using QGIS version 3.34.1 (https://qgis.org/en/site/).

## Results

**Figure 3:**
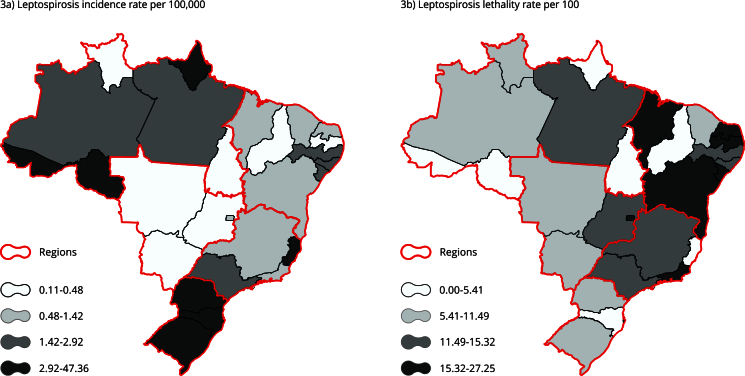
Choropleth maps of the incidence and lethality rates of human leptospirosis in Brazil from 2007 to 2019.

Note: classification by quartile. Source: Brazilian Health Informatics Department ^13^ and Brazilian Institute of Geography and Statistics ^50^.

Out of the 38,206 cases selected for analysis, 89.61% (34,238) progressed to healing, and 10.39% (3,968) resulted in death within a 90-day interval with leptospirosis as the primary cause. The overall incidence rate found was 2.72 per 100,000 inhabitants, and the lethality rate was 10.39%. The analysis of leptospirosis incidence and lethality by state revealed a distinct regional pattern (Figure 3). Generally, the states in the South and North had higher incidence rates and comparatively lower lethality, while states in the Southeast and Northeast showed lower incidence rates and higher lethality. The state of Acre recorded the highest incidence, with 47.36 cases per 100,000 inhabitants, contrasted with Santa Catarina, which had an incidence of 7.61 per 100,000. The lethality in these states was 1.11% and 3.45%, respectively. In the South, Paraná and Rio Grande do Sul had incidences of 3.33 and 4.73 per 100,000 inhabitants, with lethality rates of 11.47% and 6.02%, respectively. Meanwhile, Rondônia, Amapá, and Espírito Santo, with incidences of 3.32, 7.55, and 4.55 per 100,000 inhabitants, presented lethality rates of 4.7%, 4.92%, and 4.94%, respectively.

Deaths were more associated with urban environments (11%) than rural ones (4.8%) and were more frequent at home settings (9.8%) compared to workplaces (7.5%) or leisure locations (5.2%) (Table 1). Environmental factors, such as the presence of trash/rubble (10%) and water reservoirs (6.2%), showed a strong association with deaths. Additionally, activities like cleaning septic tanks or grease traps (9.6%) and direct contact with rodents (9.6%) also had a significant relationship with deaths. Geographically, the Southeast (14%) and Northeast (16%) had the highest proportions of deaths, while the North recorded the lowest proportion of deaths (5.7%) relative to the total number of cases.

**Table 1:** Comparison between survival and death cases from human leptospirosis in Brazil from 2007 to 2019.

Characteristics	Death (N = 3,968)	Survival (N = 34,238)	p-value *
	n (%)	n (%)	
Age (years)			< 0.001
18 to 24	389 (6.3)	5,790 (93.7)	
25 to 39	977 (7.2)	12,682 (92.8)	
40 to 49	859 (10.0)	7,452 (90.0)	
50 to 59	878 (14.0)	5,206 (86.0)	
60 to 69	563 (20.0)	2,303 (80.0)	
70 to 79	252 (27.0)	681 (73.0)	
80+	50 (29.0)	124 (71.0)	
Sex			0.046
Female	729 (9.8)	6,745 (90.2)	
Male	3,239 (11.0)	27,493 (89.0)	
Skin color			< 0.001
White	1,521 (8.2)	17,035 (91.8)	
Non-white	1,797 (11.0)	13,876 (89.0)	
Level of education (years)			< 0.001
Less than 5	456 (10.0)	3,895 (90.0)	
5 to 8	588 (7.2)	7,541 (92.8)	
9 to 11	369 (7.0)	4,901 (93.0)	
12 or older	325 (5.0)	6,207 (95.0)	
Likely infection area			< 0.001
Urban	2,552 (11.0)	20,048 (89.0)	
Rural	424 (4.8)	8,414 (95.2)	
Infection environment			< 0.001
Home	1,522 (9.8)	14,019 (90.2)	
Work	603 (7.5)	7,447 (92.5)	
Leisure	114 (5.2)	2,098 (94.8)	
Work-related illness			< 0.001
Yes	681 (7.8)	8,101 (92.2)	
No	2,072 (9.3)	20,129 (90.7)	
Cases in animals at the likely site of infection in the last two months			0.800
Yes	19 (7.9)	220 (92.1)	
No	1,745 (7.6)	21,195 (92.4)	
Floodwater or mud			0.100
Yes	1,372 (8.9)	14,019 (91.1)	
No	1,641 (8.4)	17,862 (91.6)	
Animal farming			< 0.001
Yes	716 (6.0)	11,188 (94.0)	
No	2,205 (9.9)	20,160 (90.1)	
Vacant lot			0.800
Yes	837 (8.5)	9,014 (91.5)	
No	2,047 (8.4)	22,235 (91.6)	
Trash/Rubble			< 0.001
Yes	1,212 (10.0)	10,794 (90.0)	
No	1,750 (7.8)	20,610 (92.2)	
Water reservoir			< 0.001
Yes	220 (6.2)	3,333 (93.8)	
No	2,635 (8.6)	27,846 (91.4)	
Septic tank, grease trap, or sewage system			< 0.001
Yes	637 (9.6)	6,020 (90.4)	
No	2,267 (8.2)	25,323 (91.8)	
River, stream, pond, or reservoir			< 0.001
Yes	684 (5.9)	10,879 (94.1)	
No	2,244 (9.8)	20,543 (90.2)	
Area with signs of rodents			0.700
Yes	1,939 (8.7)	20,394 (91.3)	
No	1,087 (8.8)	11,251 (91.2)	
Direct contact with rodents			< 0.001
Yes	1,051 (9.6)	9,883 (90.4)	
No	1,883 (8.1)	21,421 (91.9)	
Cultivation/Harvest (farming)			< 0.001
Yes	310 (5.1)	5,817 (94.9)	
No	2,572 (9.2)	25,406 (90.8)	
Grain/Food storage			< 0.001
Yes	225 (5.0)	4,291 (95.0)	
No	2,634 (8.9)	26,824 (91.1)	
Regions			< 0.001
North	328 (5.7)	5,446 (94.3)	
Northeast	881 (16.0)	4,796 (84.0)	
Southeast	1,815 (14.0)	10,954 (86.0)	
South	871 (6.5)	12,554 (93.5)	
Central-West	73 (13.0)	486 (87.0)	
Confirmation criteria			< 0.001
Clinical-laboratory	2,738 (8.2)	30,612 (91.8)	
Clinical-epidemiological	1,190 (26.0)	3,316 (74.0)	
Fever			< 0.001
Yes	3,303 (9.6)	31,015 (90.4)	
No	457 (15.0)	2,666 (85.0)	
Myalgia			< 0.001
Yes	3,079 (9.5)	29,278 (90.5)	
No	579 (12.0)	4,203 (88.0)	
Meningism			0.020
Yes	86 (7.4)	1,084 (92.6)	
No	3,188 (9.4)	30,902 (90.6)	
Headache			< 0.001
Yes	2,146 (7.4)	26,719 (92.6)	
No	1,369 (17.0)	6,521 (83.0)	
Prostration			< 0.001
Yes	2,378 (10.0)	20,352 (90.0)	
No	1,150 (8.5)	12,312 (91.5)	
Conjunctival congestion			0.010
Yes	643 (8.7)	6,746 (91.3)	
No	2,750 (9.7)	25,641 (90.3)	
Calf pain			< 0.001
Yes	2,053 (8.9)	20,926 (91.1)	
No	1,367 (10.0)	11,782 (90.0)	
Vomiting			< 0.001
Yes	2,067 (11.0)	17,345 (89.0)	
No	1,489 (8.7)	15,678 (91.3)	
Diarrhea			< 0.001
Yes	1,222 (10.0)	10,701 (90.0)	
No	2,227 (9.2)	22,074 (90.8)	
Jaundice			< 0.001
Yes	2,889 (16.0)	15,708 (84.0)	
No	820 (4.5)	17,354 (95.5)	
Kidney failure			< 0.001
Yes	2,039 (24.0)	6,455 (76.0)	
No	1,480 (5.4)	25,759 (94.6)	
Respiratory alterations			< 0.001
Yes	2,331 (24.0)	7,304 (76.0)	
No	1,272 (4.8)	25,205 (95.2)	
Cardiac alterations			< 0.001
Yes	662 (25.0)	1,968 (75.0)	
No	2,639 (8.1)	29,945 (91.9)	
Pulmonary hemorrhage			< 0.001
Yes	903 (27.0)	2,488 (73.0)	
No	2,462 (7.7)	29,593 (92.3)	

* Pearson’s chi-squared test.

Despite the number of confirmed cases among men being four times higher than among women, the proportion of deaths among men was only slightly higher (9.8% and 11%, respectively). The proportion of deaths increased with age and decreased with higher levels of education. Although more individuals of white skin color contracted leptospirosis (18,556 cases) compared to non-whites (15,673), non-whites had a higher proportion of death (11%) compared to whites (8.2%). A total of 26% (1,190) of the cases confirmed by clinical-epidemiological criteria resulted in death, whereas the cases confirmed by clinical-laboratory criteria were 8.2% (2,738). The variable for confirmation criteria was well-documented, with only 0.9% (350) of the data missing from the total (37,856).

Out of the total deaths (3,968), the most common symptoms present in the individuals were: fever (3,303), myalgia (3,079), jaundice (2,889), prostration (2,378), respiratory alterations (2,331), headache (2,146), vomiting (2,067), kidney failure (2,039), and calf pain (2,053). Additionally, approximately 11% of the individuals who presented with both prostration and vomiting died, along with 16% of those with jaundice, 24% of those with kidney failure and respiratory changes, 25% of those with cardiac alterations, and 27% of those with pulmonary hemorrhage.

The final model (Table 2) maintained 27 of the initial 35 variables. According to the adjustment of the final logistic regression model, the distal-level variables related to environmental characteristics that were most important for lethality included residing in an urban area (adjusted OR = 1.54; 95% confidence interval - 95%CI: 1.46-1.62) compared to rural areas, contact with trash/rubble (adjusted OR = 1.14; 95%CI: 1.10-1.18), being work-related (adjusted OR = 1.11; 95%CI: 1.05-1.19), and residing in the Southeast (adjusted OR = 1.83; 95%CI: 1.72-1.96). The least significant variable (p-value = 0.034) was direct contact with rodents when controlled for by other variables. Initially, this variable was significant (p-value = 0.001), with an 18% increase in the odds of death. This result may reflect a more immediate search for medical care by these individuals.

**Table 2:** Logistic regression analysis to determine associated factors for death from human leptospirosis in Brazil from 2007 to 2019.

Characteristics	Distal			Distal + Intermediate			Distal + Intermediate + Proximal		
	OR	95%CI	p-value	OR	95%CI	p-value	OR	95%CI	p-value
Likely infection area									
Rural	-	-		-	-		-	-	
Urban	1.80	1.72-1.89	< 0.001	1.93	1.84-2.02	< 0.001	1.54	1.46-1.62	< 0.001
Infection environment									
Home	-	-		-	-		-	-	
Work	0.77	0.73-0.81	< 0.001	0.85	0.80-0.89	< 0.001	0.88	0.83-0.94	< 0.001
Leisure	0.76	0.71-0.81	< 0.001	0.87	0.81-0.94	< 0.001	0.90	0.83-0.97	0.006
Animal farming									
No	-	-		-	-		-	-	
Yes	0.79	0.76-0.82	< 0.001	0.74	0.72-0.77	< 0.001	0.74	0.71-0.77	< 0.001
Floodwater or mud									
No	-	-		-	-		-	-	
Yes	0.94	0.91-0.97	< 0.001	0.97	0.93-1.00	0.029	0.91	0.88-0.95	< 0.001
Trash/Rubble									
No	-	-		-	-		-	-	
Yes	1.27	1.23-1.31	< 0.001	1.26	1.22-1.30	< 0.001	1.14	1.10-1.18	< 0.001
Work-related illness									
No	-	-		-	-		-	-	
Yes	1.19	1.12-1.26	< 0.001	1.14	1.08-1.20	< 0.001	1.11	1.05-1.19	< 0.001
River, stream, pond, or reservoir									
No	-	-		-	-		-	-	
Yes	0.74	0.71-0.77	< 0.001	0.77	0.75-0.80	< 0.001	0.80	0.77-0.84	< 0.001
Direct contact with rodents									
No	-	-		-	-		-	-	
Yes	1.21	1.17-1.25	< 0.001	1.18	1.14-1.22	< 0.001	0.96	0.92-1.00	0.034
Regions									
North	-	-		-	-		-	-	
Northeast	2.96	2.79-3.15	< 0.001	2.74	2.58-2.92	< 0.001	1.40	1.30-1.49	< 0.001
Southeast	2.84	2.69-3.01	< 0.001	2.75	2.60-2.92	< 0.001	1.83	1.72-1.96	< 0.001
South	1.38	1.30-1.47	< 0.001	1.40	1.31-1.49	< 0.001	1.17	1.09-1.26	< 0.001
Central-West	2.94	2.59-3.32	< 0.001	2.94	2.59-3.33	< 0.001	1.50	1.31-1.72	< 0.001
Age (years)									
18 to 24				-	-		-	-	
25 to 39				1.15	1.09-1.22	< 0.001	1.13	1.06-1.20	< 0.001
40 to 49				1.72	1.62-1.82	< 0.001	1.63	1.53-1.74	< 0.001
50 to 59				2.56	2.41-2.71	< 0.001	2.16	2.03-2.31	< 0.001
60 to 69				3.76	3.52-4.01	< 0.001	2.92	2.71-3.14	< 0.001
70 to 79				5.45	5.01-5.92	< 0.001	3.81	3.45-4.19	< 0.001
80+				5.02	4.27-5.89	< 0.001	3.69	3.05-4.46	< 0.001
Sex									
Female				-	-		-	-	
Male				1.25	1.20-1.31	< 0.001	1.10	1.05-1.15	< 0.001
Skin color									
White				-	-		-	-	
Non-white				1.20	1.16-1.25	< 0.001	1.07	1.03- 1.12	0.001
Level of education (years)									
Less than 5				-	-		-	-	
5 to 8				0.84	0.81-0.88	< 0.001	0.84	0.80-0.88	< 0.001
9 to 11				0.92	0.88-0.97	< 0.001	0.89	0.85-0.94	< 0.001
12 or older				0.72	0.69-0.76	< 0.001	0.73	0.69-0.77	< 0.001
Confirmation criteria									
Clinical-laboratory							-	-	
Clinical-epidemiological							4.17	4.00-4.35	< 0.001
Fever									
No							-	-	
Yes							0.79	0.75-0.84	< 0.001
Myalgia									
No							-	-	
Yes							0.89	0.84-0.94	< 0.001
Meningism									
No							-	-	
Yes							0.57	0.52-0.63	< 0.001
Headache									
No							-	-	
Yes							0.58	0.56-0.60	< 0.001
Vomiting									
No							-	-	
Yes							1.07	1.03-1.12	< 0.001
Conjunctival congestion									
No							-	-	
Yes							0.62	0.59-0.65	< 0.001
Calf pain									
Not							-	-	
Yes							0.71	0.68-0.74	< 0.001
Diarrhea									
No							-	-	
Yes							0.89	0.86-0.93	< 0.001
Jaundice									
No							-	-	
Yes							1.95	1.87-2.04	< 0.001
Kidney failure									
No							-	-	
Yes							2.12	2.04-2.20	< 0.001
Respiratory alterations									
No							-	-	
Yes							4.28	4.12-4.45	< 0.001
Cardiac alterations									
No							-	-	
Yes							1.63	1.55-1.71	< 0.001
Pulmonary hemorrhage									
No							-	-	
Yes							2.19	2.10-2.29	< 0.001

95%CI: confidence interval; OR: odds ratio.

At the intermediate level, related to individual characteristics, age emerged as a significant risk factor. Individuals between 50 and 59 years old had a higher risk of death (adjusted OR = 2.16; 95%CI: 2.03-2.31) compared to the 18 to 24. Being male (adjusted OR = 1.10; 95%CI: 1.05-1.15) and of non-white skin color (adjusted OR = 1.07; 95%CI: 1.03-1.12) were also associated with an increased risk of death, while a higher level of education proved to be a protective factor.

At the proximal level, related to clinical variables, jaundice (adjusted OR = 1.95; 95%CI: 1.87-2.04), kidney failure (adjusted OR = 2.12; 95%CI: 2.04-2.20), respiratory alterations (adjusted OR = 4.28; 95%CI: 4.12-4.45), cardiac alterations (adjusted OR = 1.63; 95%CI: 1.55-1.71), and pulmonary hemorrhage (adjusted OR = 2.19; 95%CI: 2.10-2.29) were identified as significant risk factors. Additionally, having the diagnosis confirmed by clinical-epidemiological criteria increased the chance of death by 4.17 times (adjusted OR = 4.17; 95%CI: 4.00-4.35) compared to those with clinical-laboratory confirmation. On the other hand, symptoms such as fever, myalgia, meningism, headache, conjunctival congestion, calf pain and diarrhea were inversely associated with lethality.

## Discussion

Our study selected 38,206 patients distributed across 2,625 Brazilian municipalities between 2007 and 2019 and revealed that approximately one in 10 patients with leptospirosis die within a 90-day interval from the onset of symptoms. Factors associated with increased lethality included age over 25 years, male, non-white skin color, lower education level, and various clinical conditions such as jaundice, kidney failure, respiratory and cardiac alterations, and pulmonary hemorrhage. Environmental factors, such as residing in urban areas and exposure to trash/rubble, were also significant. 

Little has been addressed in recent literature regarding mortality/lethality analyses of leptospirosis, and even less about the association of death with clinical and demographic variables ^1^ ^,^ ^5^ ^,^ ^6^ ^,^ ^7^ ^,^ ^22^. Articles that include environmental and animal exposure as variables mostly discuss their associations with disease incidence ^23^ ^,^ ^24^ ^,^ ^25^. This retrospective study is the first to evaluate factors associated with death in individuals with confirmed leptospirosis on a national scale, considering not only the demographic and clinical variables of the disease but also environmental exposure factors. Most research on deaths is limited to a regional scale (based on hospital data) analyzing severe cases ^22^, prolonged hospitalization due to leptospirosis ^1^, differences between pediatric and adult patients of different age groups ^26^, among other discussions.

The identification of risk factors for lethality from leptospirosis varies in the literature. Previous studies have reported pulmonary complications, such as acute respiratory distress syndrome and pulmonary hemorrhage, as well as cardiac failure, meningism, disorientation, and multiple organ dysfunction ^1^. Our results showed that fever and myalgia occurred in almost all patients, while headache, prostration, calf pain, and vomiting were reported in more than half of the patients (Table 1). In a systematic review on leptospirosis mortality conducted by Taylor et al. ^27^, these symptoms were also identified in most cases.

Additionally, the adjusted odds of death increased nearly fourfold in older people compared to younger individuals, and the odds increased by only 7% for non-white individuals. This relationship was also highlighted in previous studies, which demonstrated that older patients have a higher likelihood of death compared to younger patients ^1^ ^,^ ^7^ ^,^ ^22^ ^,^ ^28^. The proportion of deaths increased with age and decreased with higher levels of education.

Although we did not directly correlate demographic and socioeconomic factors to environmental variables, it is known that occupation, education, sex, and age are significant risk factors for leptospirosis ^29^. Moreover, according to Cataldo et al. ^30^, gender considerations continue to be neglected within the framework of *One Health*. Unfavorable environmental, sanitary, and socioeconomic conditions associated with leptospirosis exacerbate the cycles of poverty in disadvantaged communities. These conditions include contact with sewage water, accumulation of trash, and reduced rates of government sanitation interventions, such as failures in controlling rodent populations, maintaining waste collection and sewage systems, and providing universal access to clean drinking water ^31^. As highlighted by Bradley & Lockaby ^31^, these disadvantaged communities often do not receive equitable public health assistance and have been shown to face disproportionate impacts from hydrometeorological natural disasters.

Al Hariri et al. ^1^ found jaundice to be the strongest predictor for prolonged hospitalization. In the literature, jaundice and male sex have been widely associated with death from leptospirosis ^22^ ^,^ ^26^. This study also identified jaundice and male sex as predictors of death. Although the number of cases among men was four times higher than among women in this study (Table 1), the proportion of deaths between the sexes was similar (10.54% for men and 9.75% for women), and the adjusted odds of death for males increased by 10% compared to females (Table 2). Additionally, men tend to seek healthcare services less frequently or later ^32^.

The adjusted odds of death were more than four times higher in individuals with clinical-epidemiological confirmation compared to those with laboratory confirmation. The current clinical-laboratory criterion is the microscopic agglutination test (MAT), which requires the presence of antibodies after the first week of symptoms, many days after the infection. Therefore, a serological diagnosis of samples collected immediately after the onset of symptoms often leads to false-negative results ^4^. Additionally, this method is costly and time-consuming, as it is performed in reference laboratories, and often severe cases die before the results are available.

In Brazil, in endemic communities, many people do not seek medical attention, which means that many mild cases go undiagnosed and untreated. Furthermore, especially in remote areas, the population lacks access to healthcare, and diagnostic resources are limited ^27^. According to Al Hariri et al. ^1^, patients from rural areas experienced longer hospitalization periods compared to those from urban areas. The delay in seeking care, according to him, may be caused by logistical issues or a lack of awareness about the disease. This, in turn, increases the likelihood of complications and requires longer hospitalization periods.

Although our study did not analyze hospitalization duration, and our results showed that the odds of death increased by 54% in individuals residing in urban areas, the considerations raised are still relevant to our analysis. Besides logistical challenges, the lack of awareness about the disease is a significant issue in our society. Therefore, the identification and confirmation of leptospirosis are crucial tools for surveillance, public health interventions, and alerting healthcare providers ^33^.

The adjusted odds of death were more than four times higher for patients with respiratory alterations and more than twice as high for those suffering from kidney failure and pulmonary hemorrhage. Patients with cardiac alterations, jaundice, and vomiting increased their odds of death by 63%, 95%, and 7%, respectively. Although calf pain is a typical symptom that distinguishes leptospirosis from other tropical diseases, this variable was not a risk factor for death in this study. Consistent with previous research, our results align with findings from other studies. A study conducted in São Paulo from 2007 to 2016 found an incidence rate of 1.9 per 100,000 inhabitants and a lethality rate of 15.1% ^34^. Similarly, in Ceará State, a lethality rate of 12.7% was reported from 2000 to 2013 ^35^.

In Malaysia, the lethality rate found by Al Hariri et al. ^1^ was 6.48% (n = 34/525 individuals), with about one third of deaths occurring after prolonged hospitalization for more than seven days. Additionally, most deaths occurred within 24 hours of hospital admission. Factors associated with leptospirosis lethality included being male, over 40 years old, symptoms such as tachypnea, kidney diseases, multiple organ dysfunctions, respiratory failure, pneumonia, sepsis, dyspnea, pulmonary rales, pulmonary hemorrhage, rhabdomyolysis, and biochemical abnormalities ^1^. Other frequently identified symptoms were fever, myalgia, sore throat, nausea and vomiting, lethargy, abdominal pain, cough, dehydration, and jaundice ^1^.

It is estimated that the highest morbidity and mortality from leptospirosis occur in adult men aged between 20 and 49 years in resource-limited countries, as observed in South and Southeast Asia, Oceania, Caribbean, Andean, Central and Tropical Latin America, and Eastern Sub-Saharan Africa ^8^ ^,^ ^12^. Clinical studies investigating leptospirosis-related deaths have shown the presence of severe complications such as chronic pulmonary disease, diabetes mellitus, concurrent infections, hemorrhage, arrhythmia, shock, jaundice, pulmonary involvement, the need for dialysis, mechanical ventilation, and prior use of steroids ^22^. Pulmonary hemorrhage is increasingly recognized as a significant, often lethal manifestation of leptospirosis, with its pathogenesis remaining unclear ^9^. Our study identified a lethality rate of 26.63% among patients who presented pulmonary hemorrhage (903/3,391) (Table 1).

The most severe form of leptospirosis is Weil’s disease, characterized by jaundice, kidney failure, and hemorrhage, with a high lethality rate ranging from 5% to 15% ^9^. A retrospective study analyzed data collected from the emergency department of a hospital between 1989 and 1993 and identified 68 patients with positive leptospirosis serology, with a lethality rate of 18% ^5^. In Taiwan, a study with 57 confirmed cases of leptospirosis found a lethality rate of 19% ^22^. In Australia, a study found a lethality rate of 4% ^6^. In Brazil, the lethality rate found in our study was 10.38% (3,968/38,206). All regions were significantly associated with death (Table 2). The odds of death increased by 17% in the South, 40% in the Northeast, 50% in the Central-West, and 83% in the Southeast. The lowest proportion of deaths was in the North (328/5,774). Comparing these results with Figure 3b, the Northeast shows the highest number of states with high lethality. However, when examining the data by state, it is observed that the highest lethality is in the state of Rio de Janeiro, which is in the Southeast.

Despite the economic and social differences between North and South, both experience frequent flooding, which may explain the higher incidences of leptospirosis in the country. A study compiled publications that related flooding and leptospirosis in Brazil by region, finding totals of Southeast (14), Northeast (7), South (7), North (3), and Central-West (1) ^36^. Silva & Almeida ^37^ highlight the lack of research on the disease in the state of Amapá. The authors ^37^ concluded that leptospirosis is a public health problem in the state, finding an incidence of 6.6 cases per 100,000 inhabitants over 10 years (2013 to 2022), which is related to the history of low socioeconomic development in the region. Additionally, in 2019, a scarcity of supplies for leptospirosis detection by staff was reported at the Central Laboratory of Public Health (LACEN, acronym in Portuguese), responsible for the disease analysis and diagnosis in the state ^38^. In the same year, staff reported confirmed cases of leptospirosis due to a rodent infestation at the facility ^38^. 

The Tocantins State (southeast of the North Region) and the Roraima State (northwest of the North Region) had extremely low incidence rates (< 0.48), as well as the Piauí State (Northeast Region), the Paraíba State (far east of the Northeast Region), and the Central-West Region. The Tocantins State was the only Brazilian state with no reported deaths from leptospirosis between 2007 and 2019. The Piauí State reported only one death. The low incidence in the Central-West may reflect a real reduction in cases due to lower population density, delays in information reporting, underreporting, or possibly due to the clinical picture resembling other diseases, leading to underdiagnosis. Additionally, the hydrometeorological conditions in the Central-West may be favorable to the non-dissemination of leptospirosis ^11^.

Five out of the nine states in the Northeast had a lethality rate greater than 15.32%, with Sergipe State having the highest at 27.25%. The hospital lethality rate in Salvador (Bahia State) found by Lopes et al. ^26^ between 1993 and 1997 was 14.4%. During the study period (2007 to 2019), the Bahia State had a lethality rate of 17.09%. The regional differences in incidence and lethality rates for leptospirosis in Brazil from 2007 to 2019 (Figure 3) may be related to social health inequalities. According to Barata ^32^, economic, social, and political relationships affect how people live and ultimately shape the patterns of disease distribution. Thus, as important as it is to identify similarities in leptospirosis incidence from North to South, it is also crucial to consider the environmental, economic, social, and political disparities between these regions.

This finding may reflect conditions in other Brazilian states, given that the clinical forms of leptospirosis are often nonspecific or asymptomatic, contributing to its underreporting ^27^ ^,^ ^29^ ^,^ ^33^ ^,^ ^39^. Therefore, there is still a need for improved public education programs to prevent *Leptospira* infection and better disease surveillance to reduce the number of undetected cases ^40^.

In the scale we analyzed, it was not possible to extract additional variables to determine, for example, whether individuals who died had the severe form of leptospirosis, how many experienced complications, and whether those complications were resolved. This limitation arises because the notification forms are not always fully completed, and each region of the country prioritizes different aspects of data entry. The lack of data completeness in notification forms has been widely documented in studies that utilized secondary data sources ^1^ ^,^ ^7^ ^,^ ^26^ ^,^ ^34^ ^,^ ^41^.

Our study identified that deaths were more associated with urban environments than rural ones. Our hypothesis is that the serovar circulating in urban environments, *L. interrogans* serovar Copenhageni, transmitted by *Rattus norvegicus*, is highly pathogenic and associated with high lethality ^42^ ^,^ ^43^. Some serovars are more aggressive than others ^44^ ^,^ ^45^.

Furthermore, the higher number of deaths in urban environments is attributed to inadequate care for severe cases in the public healthcare system. This is due to a lack of available beds, challenges in accessing services caused by overcrowding, and missed diagnoses, as leptospirosis is often not suspected in cases without jaundice that progress to acute respiratory failure due to acute respiratory distress syndrome and pulmonary hemorrhage ^46^. Severe leptospirosis resembles bacterial sepsis in terms of clinical progression; it is well known that sepsis has a high mortality rate within the Brazilian public healthcare system ^47^ and, as demonstrated by Spichler et al. ^28^ the main risk factor for mortality in severe leptospirosis was pulmonary involvement.

Our results showed that individuals of non-white individuals had a higher proportion of death compared to white individuals. Differences in health outcomes related to skin-color have been widely described in the literature. Non-white skin color has been associated with higher mortality in other diseases, such as tuberculosis, and is linked to health inequities that reduce access to and quality of hospital treatment ^42^ ^,^ ^43^.

Regarding skin color, there is no explicit causal relationship with mortality, and studies are insufficient to assert that any racial factor could pose a risk of death. Some studies highlight a connection with socioeconomic issues, and only one study has found an association with non-white skin color ^39^. Therefore, there is no clear explanation for this finding, and further studies are needed to clarify this issue.

In the context of Public Health, it is essential to consider both the interpretation, and the magnitude of the association measures used. In this study, which analyzed factors associated with the lethality of leptospirosis, logistic regression models were employed to estimate OR, a valid measure for the analysis of this outcome. However, it is recognized that in cross-sectional studies, odds ratios can overestimate prevalence ratios, particularly when the outcome is not rare, as observed with lethality in this study. While logistic regression remains appropriate for this analysis, alternatives such as Poisson regression with robust variance have been highlighted in the literature for directly estimating prevalence ratios, offering interpretations that may be more intuitive in certain contexts ^48^ ^,^ ^49^. 

Since leptospirosis occurs worldwide, the global incidence and mortality of the disease in humans are still not well understood. Many individuals remain undiagnosed and untreated due to its nonspecific symptoms, which can be indistinguishable from other acute febrile illnesses such as dengue ^27^ ^,^ ^29^ ^,^ ^33^.
